# Database of bird species composition in natural habitats and forest plantations

**DOI:** 10.1016/j.dib.2019.104715

**Published:** 2019-10-24

**Authors:** Lucilene Inês Jacoboski, André Luís Luza, Raquel Klein Paulsen, Angelo Marcon Pezda, Sandra Maria Hartz

**Affiliations:** Laboratório de Ecologia de Populações e Comunidades, Programa de Pós-Graduação em Ecologia, Universidade Federal do Rio Grande do Sul (UFRGS), Av. Bento Gonçalves 9500, Prédio 43422, Post-Office Box: 15007, Bairro Agronomia, CEP: 91501-970, Porto Alegre, Rio Grande do Sul, Brazil

**Keywords:** Eucalyptus forest plantations, Birds, Habitat conversion, Grasslands ecosystem

## Abstract

In southeastern South America, the afforestation over grasslands imposes a severe threat to the grassy landscapes and associated biodiversity. To analyze the effect of forest plantations on grassland birds, we present a new database that considers the composition of bird communities in natural habitats, as well as in eucalyptus plantations from the southeastern South American grasslands. Data were previously used to investigate the effectiveness of legally protected grasslands in private lands to protect birds in “*Bird-grassland associations in protected and non-protected areas in the southern Brazil*” [1] and also the effects of afforestation of grasslands on different dimensions of bird diversity in “*The effects of grassland ecosystem afforestation on avian phylogenetic diversity, taxonomic diversity and evolutionary distinctiveness*” [2]. Data were collected during the breeding period of bird species (spring/austral summer), covering three breeding seasons during the years 2014–2016. Species presence and number of individuals were recorded, totaling 107 species and 1175 individuals. The dataset will be useful for researchers interested in conservation studies as it includes data from globally threatened bird species.

**Table:** Specifications Table

Subject area	*Biology*
More specific subject area	*Ecology*
Type of data	*Table*
How data was acquired	*Field sampling*
Data format	*Raw*
Experimental factors	*Incidence and number of records of bird species in native and human-modified habitats*
Experimental features	*Field sampling*
Data source location	*São Gabriel (30° 20′ 11″ S, 54° 19′ 12″W), Rosário do Sul (30° 15′* *30″ S, 54° 54′ 51″W), Santa Margarida do Sul (30° 20′ 19″S, 54°* *4′ 15″W) and Vila Nova do Sul (30° 20′ 17″S, 53° 52′ 33″W).*
Data accessibility	*Data is available with this article.*
Related research article	Jacoboski et al. “*Bird-grassland associations in protected and non-protected areas in southern Brazil*” Perspect Ecol Conserv 15, 109–114 (2017) and Jacoboski et al. “*The effects of grassland ecosystem afforestation on avian phylogenetic diversity, taxonomic diversity and evolutionary distinctiveness*” Acta Oecol 99, 103449 (2019)

**Table:** 

**Value of the Data** • Data clearly differentiate bird communities of natural habitats (forests and grasslands) and exotic forest plantations. • The dataset will be useful for managers and researchers involved in conservation policies as it includes data on birds (including threatened ones) occupying protected and managed areas [3]. • The database allows researchers to evaluate the effect of grassland afforestation on grassland birds and forest dependent birds.

## 1 Data

**Fig. 1:**
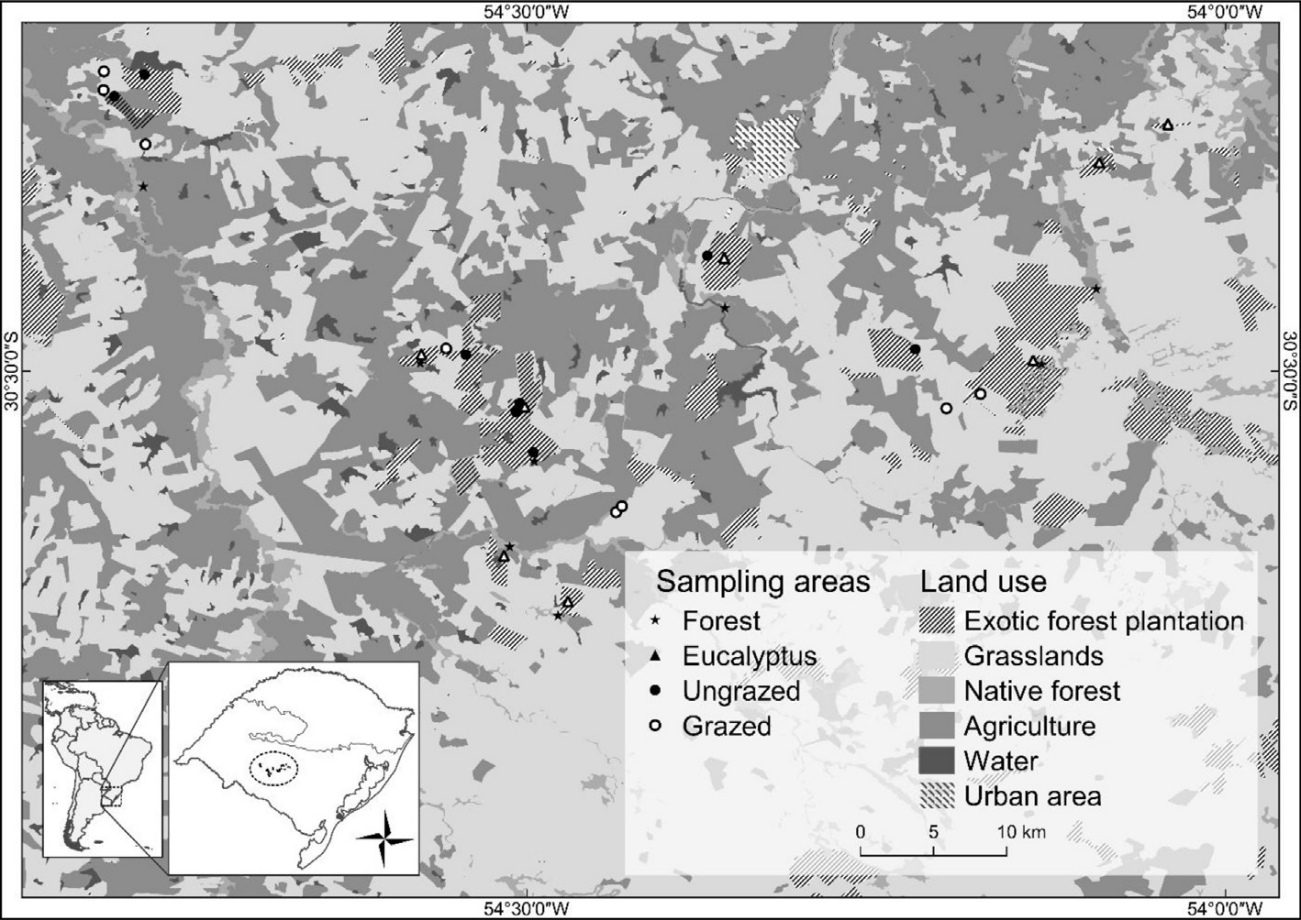
Study area map. The detail shows the location of the study area in the grasslands of Southern of the Brazil and location of the sampled sites. Each symbol represents a habitat and corresponds to sampled sites.

The data we share here is composed by recordings of bird species from natural forests, grasslands and exotic plantations of eucalyptus. The data represents the number of individuals of bird species in each of the 32 sites sampled [[1], [2]]. At the end of the study, 107 species and 1175 individuals were registered (Appendix 1). The data also contains information about the sampling sites, such as vegetation type, time and date of the sampling (Table 1). The point counting method was used to sample the birds [4]. Data were collected in the grasslands of southeastern South America (SESA grasslands), the region with the largest grassland ecosystems in the Neotropics [5] (Fig. 1). Considering the total area of the native grasslands, approximately 60% has already undergone land use changes [6]. Historically the main use of these grasslands was for livestock, but the conversion of these grasslands into silvicultural and agricultural land during the last three decades considerably modified the grassy landscape [7].

**Table 1:** Data description table. Each table row (descriptors) refers to each of the columns in the data sheet (Appendix 1). Each descriptor contains summary information from the dataset presented in the appendix (characterization of the levels). It also presents the type of descriptor and level or unit each one of them.

Descriptor	Type of descriptor	Levels/Unit of descriptors	Characterization of the levels
Habitat	Categorical	Four levels: Eucalyptus Grazed grassland Forest Ungrazed grassland	Type of habitat that bird species were registered: Eucalyptus: the eucalyptus plantations with above 15 m in height and no understory (the plantations were five to seven years old). Grazed grassland: natural vegetation with low to intermediate grazing intensity, short grassland. Forest: riparian forest with vegetation influenced by cattle and, consequently, characterized by a poorly developed understory. The vegetation ranged from six to 8 m in height. Ungrazed grassland: protecting areas known as Permanent Preservation Areas (PPAs) located within eucalyptus plantations. Within the PPAs no management (*e.g.* grazing, fire) have been performed for at least the past five years, from the time when the eucalyptus plantations were first established. Tall grassland vegetation.
Site	Categorical	32 sites sampled	Sites (localities) sampled on the region.
Point_ID	Categorical	200 count points	Each count point sampled.
Date	Categorical	One level	Date of bird species sampling.
Time	Categorical	One level	Sampling start time for each point.
Species	Categorical	One level	Identification of the bird species in each point.
Number individuals	Numerical	One level	The number of individuals of bird species in each point.

## 2 Experimental design, materials and methods

A total of 32 sites were sampled from four municipalities located in the west-central area of the state of Rio Grande do Sul: São Gabriel (30° 20′ 11″S, 54° 19′ 12″W), Rosário do Sul (30° 15′ 30″ S, 54° 54′ 51″W), Santa Margarida do Sul (30° 20′ 19″S, 54° 4′ 15″W) and Vila Nova do Sul (30° 20′ 17″S, 53° 52′ 33″W). Four different habitat types were sampled: riparian forest, ungrazed natural grassland, grazed natural grassland (all natural habitats) and eucalyptus plantations. Data were collected at eight sampling sites for each habitat type (Fig. 1).

The sample sites were selected using Google Earth images [8] and later on verified in the field. Some of the sampled riparian forests were influenced by cattle in their interior and, consequently, had poorly developed understories. The vegetation in the riparian forests ranged from six to 8 m in height. Sampled ungrazed grasslands were Permanent Preservation Areas (PPAs) located within eucalyptus plantations. The PPAs complied with the determination proposed by the Brazilian Forest Code, law 12.651/12 [9], which, among other objectives, aims to protect the vegetation along watercourse margins. Within the PPAs no management practice (*e.g.* grazing, fire) have been performed for at least five years, from the time when the plantations were first established. The PPAs that were composed mostly forest vegetation were not included. Sampled PPAs had a minimum width of 100 m [1]. For the grazed grassland sites, excessively grazed areas were not considered. These sites also had small watercourses, however, there was no maintenance of PPAs. The eucalyptus plantations considered contained trees taller than 15 m and no understory (the plantations were five to seven years old).

Birds sampling were conducted during the period of austral spring-summer from 2014 to 2016, covering three bird breeding seasons. Birds were sampled at 32 sites using the point count method [4]. The number of counts performed at each site was based on the size of the site, between three and nine point count performed at each. The point counts were separated from each other by a distance of at least of 200 m. This distance is ideal for guaranteeing statistical independence among sampled points [4]. All bird species seen or heard within a fixed radius of 50 m were recorded for a period of 10 minutes at each of the sampling points. The bird species were identified by expertise ornithologist (L.I.J). Birds in flight were not considered. The radius of observation was limited to 50 m in order to maximize detectability and decrease potential observer error, which can occur from attempting to identify cryptic species over long distances [10]. A minimum distance of 50 m was maintained from the edges of the habitat. A total of 50 sampling points were taken for each habitat type, resulting a total of 200 points. Sampling started 10 minutes after sunrise and continued for up to 3 h. All sampling was performed on days without wind or rain. The nomenclature of bird species used follows Remsen et al. [11].
